# *Phaeocystis antarctica* blooms strongly influence bacterial community structures in the Amundsen Sea polynya

**DOI:** 10.3389/fmicb.2014.00646

**Published:** 2014-12-19

**Authors:** Tom O. Delmont, Katherine M. Hammar, Hugh W. Ducklow, Patricia L. Yager, Anton F. Post

**Affiliations:** ^1^Marine Biology Laboratory, Josephine Bay Paul Center for Comparative Molecular Biology and EvolutionWoods Hole, MA, USA; ^2^Lamont Doherty Earth Observatory, Columbia UniversityPalisades, NY, USA; ^3^Department of Marine Sciences, University of GeorgiaAthens, GA, USA

**Keywords:** Amundsen Sea polynya, phytoplankton bloom, *Phaeocystis antarctica*, microbial community structure, mutualism

## Abstract

Rising temperatures and changing winds drive the expansion of the highly productive polynyas (open water areas surrounded by sea ice) abutting the Antarctic continent. Phytoplankton blooms in polynyas are often dominated by the haptophyte *Phaeocystis antarctica*, and they generate the organic carbon that enters the resident microbial food web. Yet, little is known about how *Phaeocystis* blooms shape bacterial community structures and carbon fluxes in these systems. We identified the bacterial communities that accompanied a *Phaeocystis* bloom in the Amundsen Sea polynya during the austral summers of 2007–2008 and 2010–2011. These communities are distinct from those determined for the Antarctic Circumpolar Current (ACC) and off the Palmer Peninsula. Diversity patterns for most microbial taxa in the Amundsen Sea depended on location (e.g., waters abutting the pack ice near the shelf break and at the edge of the Dotson glacier) and depth, reflecting different niche adaptations within the confines of this isolated ecosystem. Inside the polynya, *P. antarctica* coexisted with the bacterial taxa *Polaribacter* sensu lato, a cryptic *Oceanospirillum*, SAR92 and *Pelagibacter*. These taxa were dominated by a single oligotype (genotypes partitioned by Shannon entropy analysis) and together contributed up to 73% of the bacterial community. Size fractionation of the bacterial community [<3 μm (free-living bacteria) vs. >3 μm (particle-associated bacteria)] identified several taxa (especially SAR92) that were preferentially associated with *Phaeocystis* colonies, indicative of a distinct role in *Phaeocystis* bloom ecology. In contrast, particle-associated bacteria at 250 m depth were enriched in *Colwellia* and members of the Cryomorphaceae suggesting that they play important roles in the decay of *Phaeocystis* blooms.

## Introduction

Phytoplankton blooms account for a significant fraction of marine primary production. Such blooms occur in the open ocean [e.g., by the cyanobacterium *Trichodesmium* (Capone et al., 1997, 2005) or the diatoms *Hemiaulus* and *Rhizoselenia* (Subramaniam et al., 2008)] as well as in the coastal ocean (e.g., *Karenia*, *Pseudonitzschia*), where they can be a nuisance for aquaculture and fisheries. Bloom events continue to intrigue ocean researchers as the physiological underpinnings of their development, duration and demise remain unresolved (Behrenfeld and Boss, 2014). Species like *Trichodesmium* create short-lived (10–20 days) blooms of the rise-and-crash type, whereas blooms of other species may be sustained over considerably longer periods (1–3 months). The haptophyte *Phaeocystis* is a ubiquitous marine phytoplankter that causes blooms in coastal seas. Species contained in this genus have typical geographic distributions with *P. pouchetii* dominating in the Arctic Ocean, *P. globosa* in temperate coastal seas and *P. antarctica* occupying diverse niches in the Southern Ocean, respectively (Schoemann et al., 2005). *Phaeocystis* blooms significantly impact local carbon, nutrient and sulfur cycles (Van Boekel and Stefels, 1993; Yager et al., 2012) and can disturb ecosystems (Chen et al., 1999).

Extensive phytoplankton blooms occur in Antarctic waters (Arrigo and Van Dijken, 2003). In explored Antarctic polynyas (large open water expanses in sea ice), blooms are often dominated by *P. antarctica* (Arrigo et al., 1999; Smith et al., 2000; Yager et al., 2012; Kim et al., 2013). These populations are generally limited by light and iron availability (Martin et al., 1990; Bertrand et al., 2011a; Alderkamp et al., 2012) and bloom formation occurs when environmental conditions become favorable (Zingone et al., 1999; Smith et al., 2003; Vogt et al., 2012). The duration and scale of these favorable conditions are enhanced by rising temperatures and winds (Arrigo et al., 1998; Turner et al., 2005; Yager et al., 2012). *Phaeocystis* blooms occur in the surface mixed layer and they can span much of the austral summer (Arrigo et al., 1999; Wolf et al., 2013). Their populations rapidly draw down CO_2_ concentrations to <100 ppm (Arrigo and Van Dijken, 2003; Yager et al., 2012). Thus, *Phaeocystis* blooms supply organic carbon and nutrients to the food web inside polynyas (Rousseau et al., 2000; Kirchman et al., 2001a; Ducklow, 2003) and provide ecological niches for microbial heterotrophs (e.g., those capable of degrading particle organic carbon). The requirement for a continued supply of essential nutrients and growth factors like vitamins (Bertrand et al., 2011b), along with the removal of metabolites and exudates that negatively affect algal growth, likely influence bloom intensity and duration in most water bodies. The mechanisms by which *Phaeocystis* blooms sustain their activity over time are not well understood and possibly involve important functional interactions with their surrounding microbial community.

Bacteria entertain a wide range of interactions with phytoplankton (Cole, 1982; Doucette, 1995; Croft et al., 2005; Sher et al., 2011), and these interactions in turn may determine the composition of the bacterial community. A succession of bacterial taxa was observed during a phytoplankton bloom in the North Sea and their occurrence patterns were linked to their ability to degrade algal-derived organic matter (Teeling et al., 2012). The final phase of the bloom was shown to favor *Ulvibacter* and *Formosa* dominance during early and mid-stages of the decline, and to *Polaribacter* in the final stages. *Polaribacter* abundances correlate positively with chlorophyll a concentrations in the Southern Ocean and they play an active role in remineralizing organic matter generated from primary production during bloom events (Wilkins et al., 2013b; Williams et al., 2013). Not only is the free-living bacterial community affected by phytoplankton blooms, the bacterial epibionts that reside on algal cells or colonies alter their community structure as a phytoplankton bloom progresses. For example, *Trichodesmium* colonies have an epibiotic bacterial flora that is distinct from the free-living bacterial community (Hmelo et al., 2012). These changes in community structure are in part driven by chemotactic responses of bacterial taxa to phytoplankton exudates like dimethylsulfoniopropionate (Stocker et al., 2008; Seymour et al., 2010; Stocker and Seymour, 2012). Quorum sensing by associated bacteria was shown to enhance phosphate scavenging by *Trichodesmium* (Van Mooy et al., 2011).

*P. antarctica* blooms consist mostly of colonies that reach a diameter of a few millimeters and can be identified by the naked eye (Carlson et al., 1998; Smith et al., 1998). They form an important interface between the primary producers and their environment. The mucilaginous colony matrix that encapsulates the *Phaeocystis* cells forms a barrier for the exchange of dissolved compounds but it may also provide a habitat for bacterial species. Previous studies suggest that *P. antarctica* blooms affect microbial community structure in their immediate surroundings. An iron-induced phytoplankton bloom study in the Southern Ocean (West et al., 2008) showed that *Roseobacter*, SAR92 and Bacteroidetes dominated the bacterial community inside the bloom, whereas outside the bloom *SAR11*, *Polaribacter* and different *Roseobacter* types were more prevalent. A metagenomic study of coastal waters near the Antarctic Peninsula showed that bacterial communities were dominated by genotypes capable of chemotrophic, photoheterotrophic and aerobic anoxygenic photosynthetic metabolism (Grzymski et al., 2012). These communities were rich in SAR11-like genotypes, but poor in Bacteroidetes and Gammaproteobacteria (Grzymski et al., 2012). The Amundsen Sea polynya (ASP) is dominated by *Polaribacter* spp. (Bacteroidetes) and Oceanospirillales (Gammaproteobacteria) members (Ghiglione et al., 2012; Kim et al., 2013; Richert et al., submitted). These studies also reported different bacterial communities in areas with ice cover as compared to open water samples. Likewise, iceberg melt affects bacterial communities with Gammaproteobacteria dominating deep waters near icebergs and Bacteroidetes dominating elsewhere (Dinasquet et al., submitted). In accordance with findings elsewhere (Piquet et al., 2011), bacterial abundances in the ASP were highly correlated with *Phaeocystis* and diatoms suggesting a close coupling between the phytoplankton and bacterial communities (Kim et al., 2013). Bacterial productivity is not only higher in the open polynya as compared to adjacent water bodies, the bulk of bacterial exoenzyme activity, respiration and production was associated with the size fraction that contains *Phaeocystis* particles (Williams et al., submitted). However, so far no efforts have been made to assess whether members of the bacterial community interact directly with *Phaeocystis*. We hypothesized that the bacterial community is not limited to the biomineralization of organic carbon and nutrients but that specialized members of this community may also entertain interactions with *Phaeocystis* that stimulate bloom formation or act to enhance and perpetuate such blooms.

In an attempt to investigate the occurrence of such interactions we sampled two *P. antarctica* bloom events (2007–2008 and 2010–2011) from the highly productive ASP in west Antarctica (Arrigo and Van Dijken, 2003; Alderkamp et al., 2012; Mills et al., 2012). We targeted the V6 hypervariable region of the 16S rRNA gene with primers that target bacterial and eukaryotic organelle templates. We used this approach to generate large V6 sequence datasets (10^5^–10^6^ reads per sample) for various locations at the shelf break, inside the polynya and near the Dotson glacier. We coupled the depth and quality of paired-end Illumina sequencing to oligotyping, a sensitive bioinformatics tools to partition conserved genotype clusters within key microbial taxa, revealing an extended diversity. Using different sampling strategies, we discovered a number of bacterial taxa that preferentially associate with *P. antarctica* and their abundance correlated with that of *Phaeocystis*. We also identified different bacterial taxa that may play a specific role in bloom demise and the degradation of *Phaeocystis* biomass at depth.

## Materials and methods

**Figure 1:**
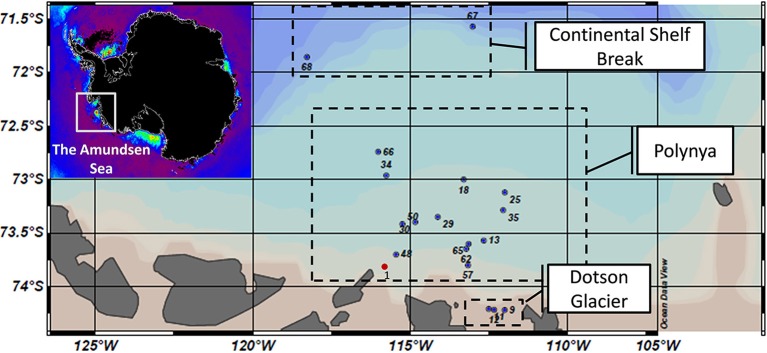
**Map of sampling sites across the Amundsen Sea polynya that were visited during the Oden Southern Ocean (OSO) cruise in 2007–2008 (red dot) and the ASPIRE cruise in 2010–2011 (blue dots)**. Samples were grouped into three major regions: the Continental Shelf Break, the open waters of the polynya and waters abutting the Dotson Glacier. Insert: A map of chlorophyll *a* concentrations (red: high; purple: low) in polynyas abutting the Antarctic continent as estimated by remote sensing (courtesy of Dr. Kevin R. Arrigo, Stanford University). Note that sample numbers are only informative of the station number tracked during the ASPIRE cruise. The corresponding samples are presented in the supplemental material.

Water samples were collected at various sites across the ASP during the austral summers of 2007–2008 (aboard the icebreaker R/V “Oden”; depth profiles) and 2010–2011 (ASPIRE cruise aboard the R/V “Nathaniel B Palmer,” horizontal grid of surface samples) (Figure 1, Table S1). Cruise track, sampling sites and an overview of geochemical and biological properties have been detailed elsewhere (Yager et al., 2012). Additional information can be found in the BCO-DMO database (http://osprey.bco-dmo.org/project.cfm?id=146&flag=view) and in Table S2. For the 2010–2011 cruise, water samples (3–10 L) for microbial community sequence analyses were passed over a 20 μm mesh and collected onto 0.2 μm Sterivex membrane filter cartridges by pressure filtration (Whatman Masterflex L/S series). Since high biomass caused rapid clogging of the filters, the sampling volumes varied between stations. Two distinct plankton size classes (0.2–3 μm and 3–200 μm) were fractionated for samples collected during the 2007–2008 cruise. This sampling effort (10–20 L) was done along a depth profile that spanned the full water column (Figure 1, Table S1) and the microbial community analysis was part of the International Consensus for Marine Microbes project. Filters were quickly frozen in the headspace of a LN_2_ Dewar and stored at −80°C prior to DNA extraction. We note that the 2007–2008 data were determined on samples from a single depth profile inside the ASP. It was decided to incorporate these data in order to derive first hints regarding the reproducibility of bacterial community compositions that accompany *Phaeocystis* blooms in the ASP and to gain early insights into the bacterial taxa that may associate with *Phaeocystis* colonies and other particles. Metadata of the various samples are presented in Table S1 and in the supplemental material. DNA extraction was performed using the Puregene kit (Gentra®) after disruption of the cells with lytic enzyme coupled to proteinase K (Sinigalliano et al., 2007). DNA concentrations were quantified using a Nanodrop 2000 instrument (Thermo Fisher Scientific, Wilmington, DE).

The V6 hypervariable region of the 16S rRNA gene (typically 60–65 bp in length) was amplified (25 cycles using HiFi buffer 1X, MgSO_4_ 2 mM, dNTPs 0.2 mM, combined primers 0.2 mM and four units of platinum HiFi) in triplicate PCR reaction from 10 ng of environmental DNA templates with reverse primer (1046R) “CGACRRCCATGCANCACCT” and the forward primer mix (967F) “CTAACCGANGAACCTYACC,” “ATACGCGARGAACCTTACC,” “CNACGCGAAGAACCTTANC,” and “CAACGCGMARAACCTTACC.” PCR cycle conditions were defined as follow: 30 s at 94°C followed by 45 s at 60°C and 1 min at 72°C. The PCR started with 3 min at 94°C and ended with 2 min at 72°C followed by a rapid stepdown to 4°C. Negative controls (no template DNA) were run for each of the index primer combinations in the PCR reactions. V6 amplicon sequences from samples collected during the 2007–2008 R/V “Oden” cruise (*n* = 12) were obtained on a GS-FLX pyrosequencing platform. Sequence reads were subsequently trimmed for low-quality sequences (Huse et al., 2007). For samples collected on the ASPIRE cruise during the 2010–2011 austral summer (*n* = 23), a paired-end sequencing strategy for Illumina Hiseq platform was employed with custom fusion primers described previously (Eren et al., 2013b) targeting the V6 hypervariable region of the 16S rRNA gene. The library design provided a complete overlap of the V6 region, and high-quality V6 reads were generated by requiring a complete match between the two reads of each pair (Eren et al., 2013b). Read sizes of the trimmed datasets are presented in Table S1.

Quality-filtered datasets were subsequently annotated using the Global Assignment of Sequence Taxonomy (GAST) pipeline (Huse et al., 2008) using the SILVA 111 database for reference (Quast et al., 2013). The datasets are publically accessible through the VAMPS website (http://vamps.mbl.edu/) under the project names ICM_ASA_Bv6 (2007–2008) and AFP_ASPIR_Bv6 (2011–2012). In order to assess within taxon diversity, reads affiliated to a given genus with GAST were submitted to oligotyping, a computational method for taxonomical partitioning based on Shannon entropy decomposition (Eren et al., 2013a). By utilizing only the nucleotide positions that show high variation, and disregarding the redundant sites with low entropy, oligotyping analysis employs only a fraction of the nucleotide positions across the read length, hence reduces the impact of random sequencing errors while maintaining high sensitivity to discriminate closely related but distinct taxa. STAMP software (Parks and Beiko, 2010) was used to observe taxonomical structure variations inferred from 16S rRNA gene amplicon datasets. Furthermore, principal component and correspondence analyses were performed using the “R” and Ade4TkGUI software packages (Thioulouse et al., 1997). Box plots were generated using R. A Pearson statistical test was used to study the correlation of specific microbial taxa among the datasets. Finally, One-Way ANOVA tests were used to access the significance of community structure shifts observed between groups of samples.

## Results

### Distant locales in the southern ocean have distinct microbial communities

During the 2010–2011 austral summer, surface water samples were collected at 18 sites (plus 5 near-bottom water samples for a total of 23 samples) across the ASP (Figure 1): 3 sites along the Dotson glacier, 2 sites covered by pack ice at the outer fringe of the polynya near the shelf break and 13 sites across a bloom of *Phaeocystis antarctica* (>10 μg Chl *a*.L^−1^, O_2_-saturation at >400%, pCO_2_ 100–250 ppm) in the open waters of the polynya. Bacterial community structures (0.2–20 μm) were determined by deep sequencing of 16S rRNA amplicons [60–65 nucleotides of the V6 hypervariable region, >10^5^ reads per sample (Table S1)]. Even though our primer sets amplified some Archaea V6 (and thus provided an approximation of their diversity) we did not include these data here. The diversity and low abundance of Archaea in the ASP center (Archaea/Bacteria ratio of about 1/500) was part of a separate study (Kim et al., 2013). Our observations on the microbial community structures focused on the diversity, relative abundance and distribution of bacterial and eukaryotic taxa.

In a first approach we compared the bacterial communities of four randomly selected ASP surface samples [Ant11, Ant13, Ant14, Ant15 (Table S1)] with those determined for four surface water samples from the Antarctic Circumpolar Current (ACC) and four samples near the Antarctic Peninsula (Sul et al., 2013). Figure S1 shows a heat map comparison of these different sites. It is immediately apparent that the microbial community structures had a high degree of similarity within datasets for each of the three locales in the Southern Ocean, but substantial dissimilarity was noted between datasets. The bacterial communities in the ASP were most dissimilar from those at the two other locales. The Bray-Curtis dissimilarity index for samples inside the ASP was only 0.27 ± 0.13. However, the dissimilarity index increased substantially when comparing different locales: 0.54 ± 0.06 between ASP and ACC; 0.64 ± 0.04 between ASP and waters off the Antarctic Peninsula.

**Figure 2:**
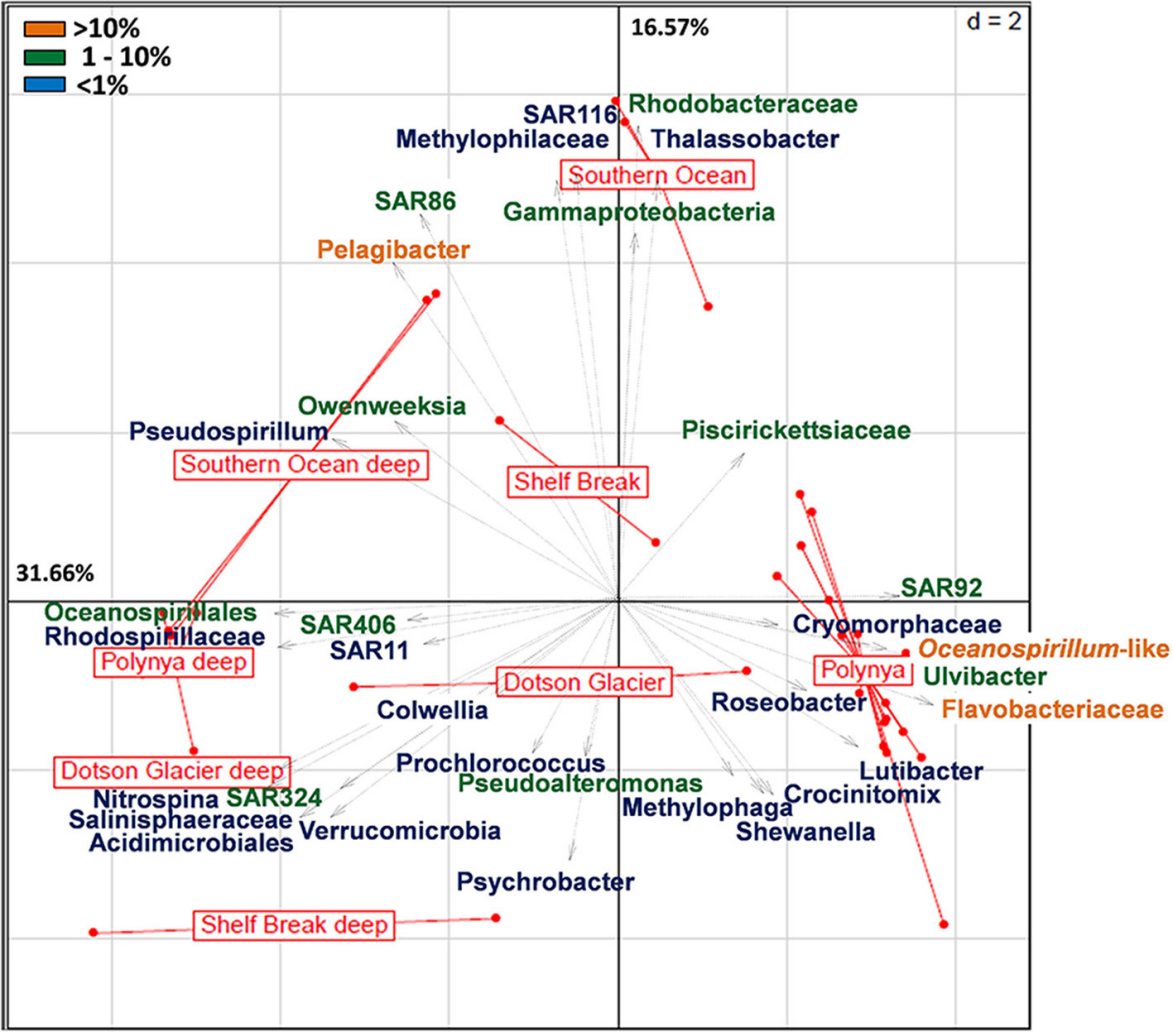
**Principal component analysis (euclidean distance) of the relative distribution of the 36 most abundant bacterial taxa across high throughput sequencing datasets from the Southern Ocean (Antarctic Circumpolar Current) and the Amundsen Sea (shelf break, Dotson glacier and polynya)**. The latter includes surface and deep bacterial communities sampled at the outer rim of the polynya, near the Dotson Glacier and in the open waters of the polynya. Pyrosequencing datasets of the Amundsen Sea polynya representing the 0.2–3 μm size fraction sampled during the 2007–2008 bloom event (4 surface and 3 deep samples; see Table S1) were included in the analysis. Orange labels denote taxa that contribute >10% of the bacterial community; green denotes 1–10%; Blue is <1%.

A more detailed visualization of the difference among the bacterial communities from the three locales is presented in Figure 2. Whereas the deep samples (most diverse) populated the left two quadrants of both the Principal Component Analysis (PCA) and Correspondence Analyses plots, surface samples (less diverse) organized along the central axis with the exception of the samples that represent the surface waters in the center of the polynya where the *P. antarctica* bloom occurred (Figure 2, Figure S2). The latter samples all grouped together in a cluster distant from the other samples. The axes in the PCA plot account for about 48% of the total variance. These differences were mainly driven by a dominant contribution of *Pelagibacter* (>10% of total), SAR86, members of the Gamma-Proteobacteria and *Rhodobacteraceae* (each at 1–10%) in the ACC samples. In contrast, bacterial communities in surface waters of the central polynya were dominated by a large group of Flavobacteria, Oceanospirillales, SAR92 and *Ulvibacter* (>1% total) with a minor but significant contribution (<1%) made by *Lutibacter*, *Crocinotimix*, *Roseobacter* and members of the Cryomorphaceae. The microbial community that accompanied the *P. antarctica* bloom was different from those near the Dotson glacier or at the outer fringes of the polynya near the shelf break.

### Microbial community structure inside the amundsen sea polynya

**Figure 3:**
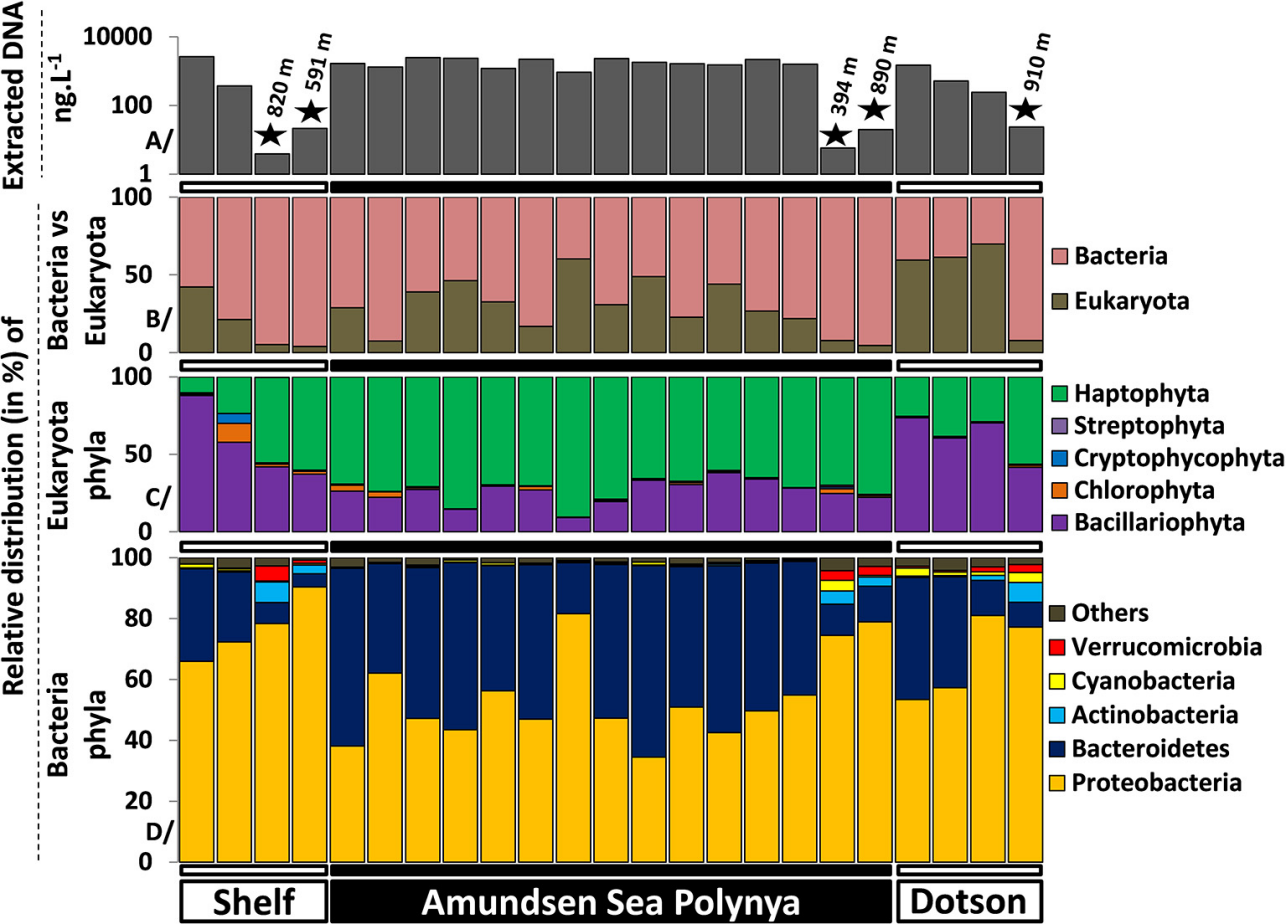
**Microbial community structures determined by Illumina sequencing (>10^5^ reads per sample) of 16S-V6 rRNA amplicons obtained from surface and deep samples at sites with dense ice cover at the shelf break (Shelf), inside the Amundsen Sea polynya and in open waters adjacent to the Dotson glacier (Dotson) during a *Phaeocystis antarctica* bloom in 2010–2011**. **(A)** (top) denotes the concentration of extracted DNA for each sample as a proxy for microbial biomass; the “★” symbols denote deep (>350 m) samples. The relative contribution of Bacteria vs. Eukaryota (chloroplast 16S) is presented in **(B)**. **(C,D)** present the phytoplankton and bacterioplankton community composition at the phylum level.

To better understand differences in microbial communities at different locations in the Amundsen Sea we analyzed the taxonomic composition of Eukarya and Bacteria across the polynya. On average, 1.58 ± 0.72 and 0.015 ± 0.009 μg L^−1^ of DNA were extracted from surface and deep samples, respectively. DNA yields were two orders of magnitude lower in the deeper samples, reflecting the lower biomass levels of this size fraction at depth (Figure 3A). Eukarya (>99% phytoplankton taxa) were an important fraction in surface waters (Figure 3B), most prominent near the Dotson glacier (62 ± 5.8%). Bacteria made up the bulk of the community in deep samples (94 ± 1.8%) where the number of detected species (576 ± 131) significantly increased (*p* < 0.001) in comparison to surface samples (361 ± 51) (Figure S3). Among the Eukarya, haptophyte (>99% *Phaeocystis antarctica*) genotypes dominated the phytoplankton bloom in the ASP (72 ± 7.8%) while diatoms (*Bacillariophyta*) were more abundant near the Shelf break and the Dotson glacier (70 ± 12%, see Figure 3C). Note that 16S rRNA gene copy number can vary widely between alga species depending on the number of chloroplasts per cell. Therefore, the ratio of Bacteria/Eukarya and haptophyte/diatoms in each dataset are not necessary representative of the plankton community structure. On the other hand, ratio differences observed for the same populations between samples are more likely to reflect shifts in community structure. In particular, we observed a clear shift from a Proteobacteria dominated bacterial community (73 ± 11.2% vs. 50 ± 8.3%, *p* < 0.001) outside the *P. antarctica* bloom to a Bacteroidetes dominated community (47 ± 8% vs. 18 ± 13.1%, *p* < 0.001) inside the bloom. The abundant *Phaeocystis* populations in central waters of the polynya were accompanied by a bacterial community dominated by Flavobacteria (99 ± 0.8% of total Bacteroidetes), and Proteobacteria. Together they contributed >95% of the V6 reads in each of the polynya samples (Figure 3D). Inside the polynya the Proteobacteria were dominated by Gammaproteobacteria (73 ± 6.2%, mostly *Oceanospirillum*-like and SAR92) and Alphaproteobacteria (24 ± 6.2%, mostly *Pelagibacter*) with lesser contributions made by Betaproteobacteria (1.2 ± 1.1%, mostly *Methylophilaceae*), Deltaproteobacteria (0.7 ± 0.4%) and few Epsilonproteobacteria (0.0006 ± 0.004%). We also explored a dataset from another bloom event (R/V Oden cruise, 2007–2008) that was sampled along a single depth profile (Table S1) and we compared their microbial communities. Four samples that originated from the upper 100 m of the water column and that had Chl a concentrations of >8 μg L^−1^ all revealed microbial community structures that were highly similar to those of the 2010/2011 bloom (see Figure 2).

In contrast to the surface samples the bacterial communities in deep samples (Figure 3D) were characterized by an increased contribution of Proteobacteria (80 ± 6.1%) and decreasing Bacteroidetes abundances (8.3 ± 2.9%). Both the increase in the prevalence of Proteobacteria and the decrease in Bacteroidetes abundance were significant (*p* < 0.01) in One-Way ANOVA tests. Verrucomicrobia (3.0 ± 1.4%) and Actinobacteria (4.7 ± 1.8%), typically found in deep marine waters (Sogin et al., 2006; Quaiser et al., 2008; Freitas et al., 2012), were significantly more abundant in deep samples (Figure 3D). The Proteobacteria were dominated by Gammaproteobacteria (47 ± 14.6%, mostly *Pseudoalteromonas* and *Oceanospirillales*), Alphaproteobacteria (33 ± 15.6%, mostly *Pelagibacter* and *SAR11* related taxa) and Deltaproteobacteria (19 ± 5.7%, mostly *SAR324* and Nitrospina) with a minor contributions made by Betaproteobacteria (0.4 ± 0.3%) and Epsilonproteobacteria (0.2 ± 0.1%) classes. The Delta and Epsilon classes were therefore drastically more represented in deep samples. Three samples from 250 to 785 m depth within the polynya water column and collected during the 2007–2008 bloom event provide similar trends (these samples are part of the “Polynya deep” group in Figure 2), with a dominance of *Pelagibacter*, *Oceanospirillales* and *SAR324* genotypes. Altogether, our findings indicate that the surface and deep microbial community structures of the Amundsen Sea polynya were highly similar across the spatial dimensions of the bloom. They were also maintained across temporal scales that exceed bacterial generation times by far. We note that these highly similar bacterial communities were maintained over a 18 day period (19/12/2010 to 05/01/2011), close to the climax of the ~90 day bloom duration estimated from remote sensing images (Arrigo and Van Dijken, 2003).

### Oligotype diversity of bacterial and eukaryotic taxa

**Figure 4:**
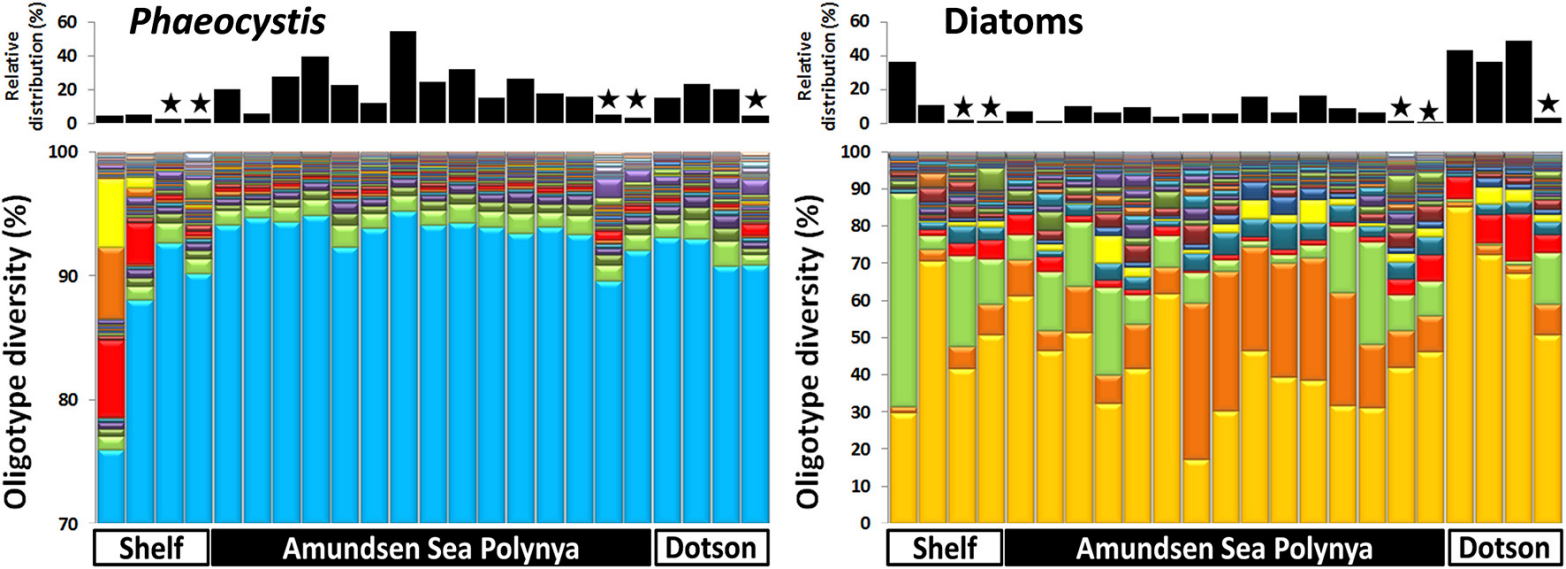
**Phytoplankton oligotype diversity of 16S-V6 rRNA amplicon sequences obtained for *Phaeocystis* (left graph) and diatoms (right graph)**. Surface and deep samples were obtained at sites with dense ice cover at the shelf break (Shelf), inside the Amundsen Sea polynya and in open waters adjacent to the Dotson glacier (Dotson) during a *Phaeocystis antarctica* bloom in 2010–2011. Panels at top of the graphs denote the relative contribution of a taxon within each dataset; the “★” symbols denote deep (>350 m) samples. Oligotypes detected at low abundance (*n* < 200) in the dataset were removed from the analysis.

**Figure 5:**
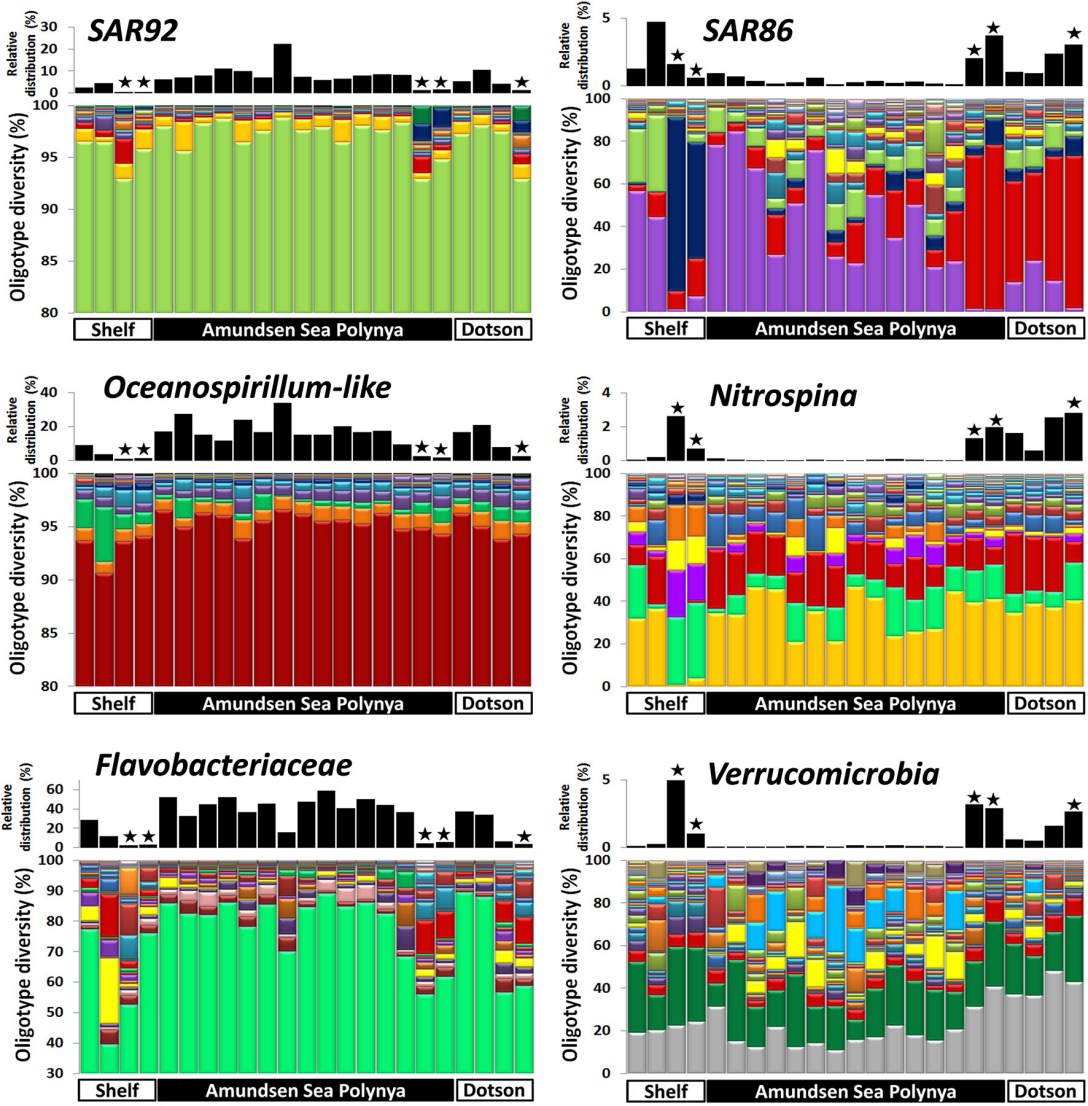
**Bacterial oligotype diversity of 16S-V6 rRNA amplicon sequences obtained for taxa that dominated inside (left graphs) and outside (right graph) the *Phaeocystis* surface bloom**. Surface and deep samples were obtained at sites with dense ice cover at the shelf break (Shelf), inside the Amundsen Sea polynya and in open waters adjacent to the Dotson glacier (Dotson) during a *Phaeocystis antarctica* bloom in 2010–2011. Panels at top of each graph denote the relative contribution of a taxon within each dataset; the “★” symbols denote deep (>350 m) samples. Oligotypes detected at low abundance (*n* < 200) in the dataset were removed from the analysis.

**Figure 6:**
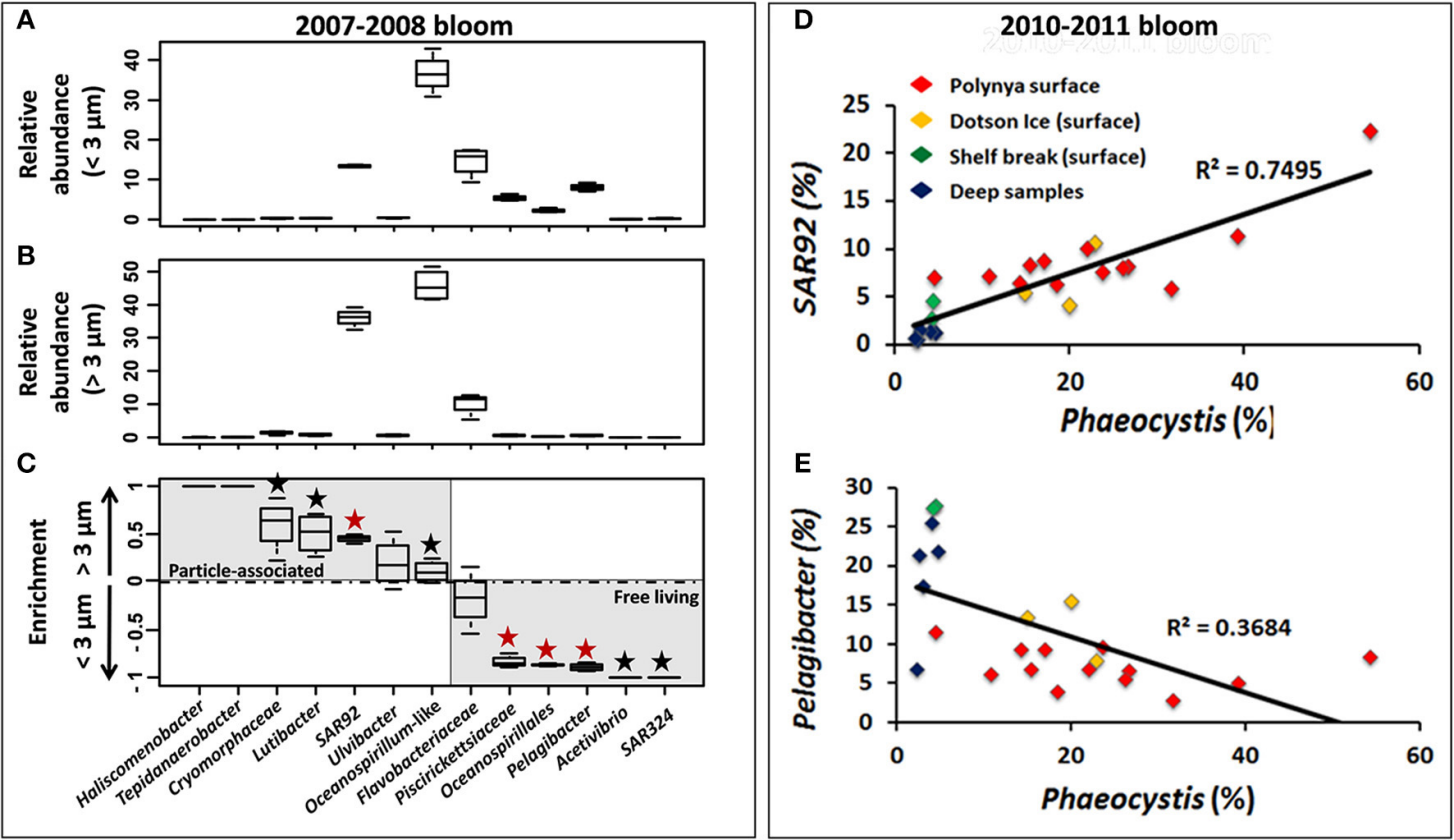
**Relative abundance of 13 bacterial taxa in the <3 μm (A) and >3 μm (B) size fractions and preferential enrichment (C) of these taxa in either fraction among *Phaeocystis* bloom samples from surface waters in the Amundsen Sea polynya in 2007–2008**. ANOVA test was performed using STAMP software to test the significance of their enrichment in the two size fractions. For each taxa, a black “★” symbol was added when *p*-value score was lower than 0.05 (>95% confidence level). This symbol displayed in red indicates a *p* < 0.01 (>99% confidence level). Correlation between *Phaeocystis* genotypes (percentage of the whole community) and SAR92 **(D)** or *Pelagibacter* **(E)** (percentage of the bacterial community) are displayed across all datasets during the *Phaeocystis* bloom of 2010–2011.

In order to analyze the bacterial communities in more detail we studied the diversity of selected taxa across the polynya during the 2010–2011 bloom event (Figures 4–6). Shannon entropy decomposition or oligotyping (Eren et al., 2013a) was used to track subtle, conserved sequence variations and differentiate genotypes that make up each taxon but differ by as few as 1–7 nucleotides. Among the chloroplast V6 reads, those identified as *Phaeocystis antarctica* were dominated by a single oligotype (>90%) at all locations and depths inside the polynya (Figure 4). A few different oligotypes were distinct in surface waters near the shelf break, suggesting the existence of sub-populations of *P. antarctica* that do not contribute to bloom formation. Diatom populations were more diverse (Figure 4). Among the bacterial V6 reads, the major taxa in surface layers of the polynya were dominated by a single oligotype for SAR92 (97 ± 1.8% of total), *Oceanospirillum*-like bacteria (95 ± 1.4%), *Pelagibacter* (80 ± 7.3%, data not shown) and members of the Flavobacteriaceae (75 ± 14.1%) a family for which V6 sequence do not allow taxonomy assignment below the rank of family (Figure 5). These taxa showed different oligotype diversity patterns in deep samples with the exception of the *Oceanospirillum-like* oligotype diversity that remained strikingly similar despite the strong variation in their relative abundance (0.9–33.9% of the bacterial community) across the datasets. The dominant oligotype related to Flavobacteriaceae could not be resolved taxonomically due to a perfect match between V6 sequences of *Polaribacter* and other members of the Flavobacteriaceae. This oligotype was therefore denoted as *Polaribacter* sensu lato. On the other hand, taxa such as SAR86, *Nitrospina* and *Verrucomicrobia* that were detected in higher abundance outside the bloom (especially in deep samples) were more diverse and lacked a single dominant representative oligotype for either of the niches (Figure 5). For SAR86, one oligotype dominated the deep samples while another oligotype was more abundant in the surface waters at the shelf break. The two oligotypes represent bacteria that have so far evaded successful cultivation. *Nitrospina* was equally diverse in all samples except at the shelf break where we observed a few distinct oligotypes. Finally, the diversity among Verrucomicrobia genotypes appeared to be relatively uniform across all datasets with minor variations observed between different locales.

### Free living vs. particle associated bacterial taxa

During the 2007/2008 bloom event in the Amundsen polynya samples from the surface (above 100 m depth, *n* = 4) and at 250 m depth (*n* = 1) were size fractionated. The size fractionation differentiated between bacteria in the 0.2–3 μm (<3-μm) and 3–200 μm (>3-μm) fractions. The <3-μm fraction was thought to be enriched with V6 reads from free-living bacteria, whereas V6 reads in the >3-μm fraction were derived from particle-associated bacteria. The most common particles were *Phaeocystis* solitary cells and colonies along with diatom species that together gave rise to the intense phytoplankton bloom. We observed a substantial increase of *P. antarctica* (from 25 ± 17.1 to 51.2 ± 24.3% of total V6 reads) and a much less pronounced increase in diatom V6 reads (from 1.7 ± 0.8 to 9.5 ± 4%) in the >3-μm fraction of the polynya surface. Presumably, *P. antarctica* was present as single cells in the <3-μm fraction and as small-medium sized colonies in the >3-μm fraction. A taxon-by-taxon comparison of bacterial genotypes between the two size fractions in the surface mixed layer of the polynya identified several taxa that showed a higher relative abundance in either <3-μm or >3-μm fractions (Figures 6A–C). For each taxon we calculated the relative enrichment as the ratio of their abundance in the two size fractions using the following equation:

(1) $$\left(\frac{2\text{X }(>3\;\mu \text{m fraction})}{<3\;\mu \text{m fraction }+\;>3\;\mu \text{m fraction}}-1\right)$$

A PCA showed that a large majority of dominant taxa were enriched in the small fraction (Figure S4). SAR92 reads were abundant in each fraction but this genus was significantly enriched in the >3-μm fraction. Whereas they contributed ~13% of the V6 reads in the <3-μm fraction their contribution rose to >35% in the >3-μm fraction (Figures 6A,B). Thus, the enrichment ratio of SAR92 (Gammaproteobacteria) was close to 0.5 (Figure 6C). Similarly, *Oceanospirillum*-like (Gammaproteobacteria) genotypes were abundant in both the <3-μm and >3-μm fractions, but their enrichment was less pronounced (Figure 6C). Several low abundance taxa – a single Firmicute,* Tepidanaerobacter*, and Bacteroidetes genotypes identified as* Haliscomenobacter, Lutibacter*, *Ulvibacter*, and *Cryomorphaceae* showed the same trend as was observed for SAR92 and they were enriched in the >3-μm fraction (Figure 6C). In contrast, different *Flavobacteria*, *Oceanospirillales*, and *Piscirickettsiaceae* (Gammaproteobacteria), and *Pelagibacter* (Alphaproteobacteria) together with the low abundance taxa *Acetivibrio* (Firmicutes) and *SAR324* (Deltaproteobacteria), dominated in the <3-μm fraction and they were presumably present mostly as free-living cells (Figures 6A–C).

A similar picture of an association of certain bacterial taxa with *Phaeocystis* particles emerged from a further analysis of the 2010/2011 bloom event in the ASP. We observed a significant correlation (*R*^2^ = 0.75, *p* = 9.5e-8) between the relative abundances of *P. antarctica* and those of SAR92 across 23 datasets (Figure 6D). Note that relative abundances of SAR92 were normalized to the number of bacterial reads in each sample whereas relative abundances of *Phaeocystis* were normalized to total reads (bacteria + chloroplast reads). Assuming a single 16S rRNA gene copy per SAR92 genome (as detected in SAR92 HTCC2207) and the presence of two chloroplasts per alga cell (Moisan et al., 2006), each with two single 16S rRNA gene copy (as detected in the chloroplast genome of *P. antarctica* strain CCMP1374) we estimate an approximate 2:1 occurrence of SAR92 per *Phaeocystis* cell. On the other hand, *Pelagibacter* V6 reads, which were more abundant in the <3-μm fraction (Figure 6C), showed a trend of decrease (while not significant) with increasing abundance of *Phaeocystis* (Figure 6E), thereby independently confirming the observations made for the 2007/2008 bloom event.

**Figure 7:**
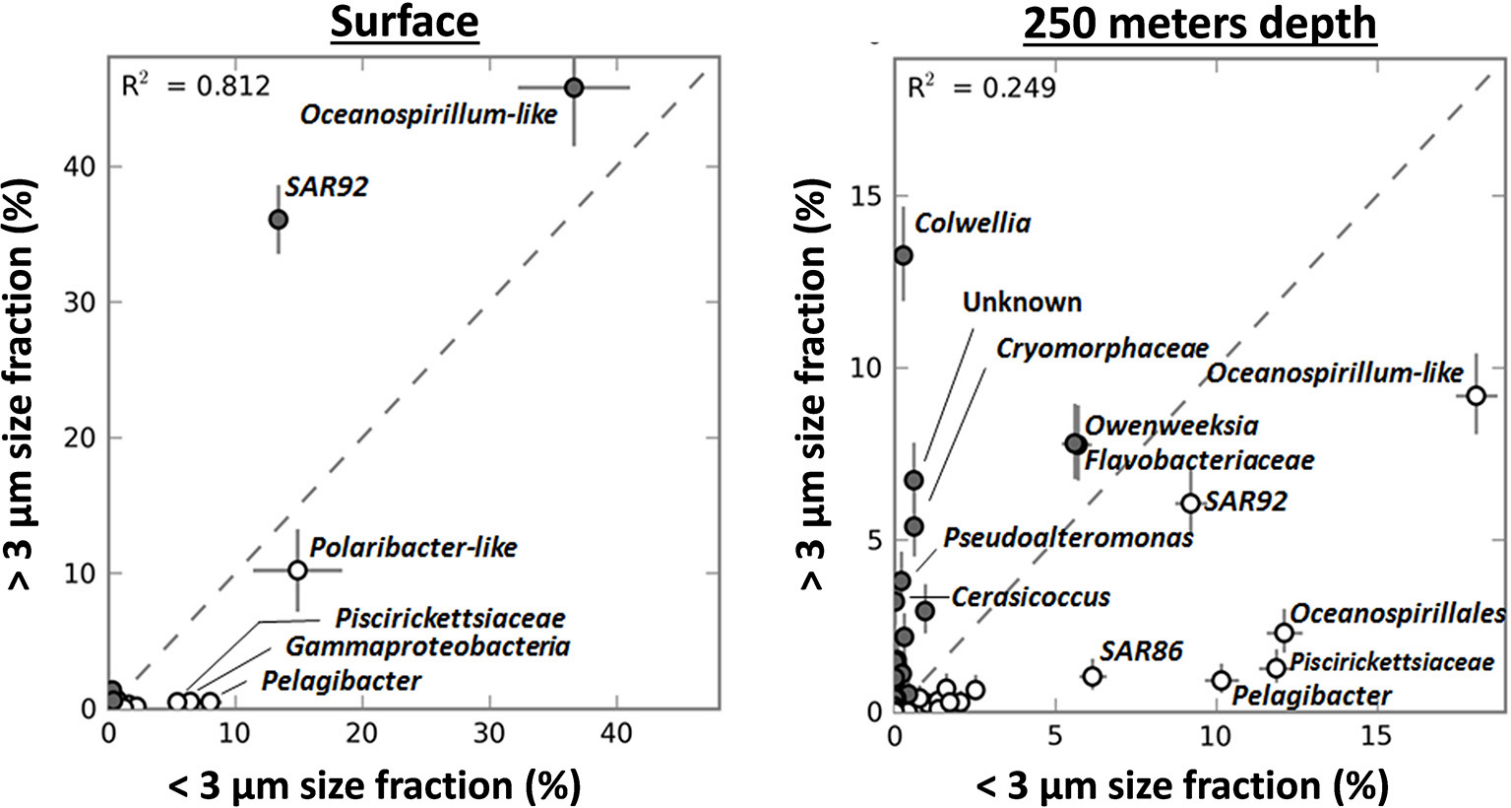
**The relative distribution in percentage of genera (based on GAST classification and using STAMP software) in the <3 μm and >3 μm size fractions in samples from surface layers (*n* = 8) and from 250 m depth (*n* = 2) during the *Phaeocystis* bloom in 2007–2008**. The coloring of circles reflects the enrichment of the taxa (closed circles when more abundant in the >3 μm size fraction, open circles when more abundant in the <3 μm size fraction).

In contrast to their relative abundances in the surface samples, diatoms were more enriched in the >3-μm fraction of the deep sample taken during the 2007/2008 bloom (from 0.8 to 56.6% of the total V6) than *P. antarctica* (from 7 to 21.8%) (data not shown). V6 reads for taxa such as *Pelagibacter*, Oceanospirillales and Piscirickettsiaceae were still more abundant in the <3-μm fraction. However, strong shifts in taxonomic make-up of the >3-μm fraction as compared to the <3-μm fraction occurred (Figure 7). SAR92 V6 reads were not enriched in the >3-μm fraction of the sample taken from below the surface mixed layer. Although by no means significant, its relative abundance was slightly higher in the <3-μm fraction (+3.2% of the bacterial community). However, in addition to the omnipresent *Cryomorphaceae* and* Ulvibacter* other taxa became more enriched in the >3 μm fraction. In particular we identified *Colwellia*, *Pseudoalteromonas* and *Cerasicoccus* genotypes that were associated with decaying *Phaeocystis* and/or diatoms, the dominant type of particles in samples below the surface mixed layer. The relative enrichment of these taxa in the >3-μm fraction [as compared to the <3-μm fraction, see Equation (1)] was by a ratio of 0.96 (*Colwellia*), 0.90 (*Pseudoalteromonas*) and 0.98 (*Cerasicoccus*). These relative enrichments were much less pronounced in surface samples with ratios of 0 (±0.09), 0.24 (±0.35), and 0.12 (±0.38) respectively.

## Discussion

Whereas persistent blooms of *Phaeocystis antarctica* have been reported for multiple Antarctic polynyas (Arrigo et al., 1999; Smith et al., 2000; Arrigo and Van Dijken, 2003; Alderkamp et al., 2012; Yager et al., 2012) and even in the ACC proper (Alderkamp and van Dijken, pers. comm.), we do not understand all the factors that drive bloom formation and/or support bloom longevity. Previous studies have focused on Fe-limitation of such blooms (Mills et al., 2012) and the role of Fe-supply from glacier melts to polynya surface waters (Alderkamp et al., 2012). Other studies addressed the role of *Phaeocystis* colony formation and control of colony size by grazer populations (Tang et al., 2008). Here we studied the potential for the bacterial flora to play a role in the bloom biology of *P. antarctica*. We have obtained the deepest sequencing of the ASP to date: 10^5^–10^6^ paired-end (100% overlap) reads for the V6 hypervariable region of 16S rRNA per sample as compared to other studies that report 10^3^–10^4^ reads for V1 and V3–V4 obtained by pyrosequencing (Kim et al., 2013; Dinasquet et al., submitted; Richert et al., submitted). The complete overlapping sequencing strategy performed here enhanced sequence quality for each V6 read, and so provided highly reliable signatures for the detection of low abundance bacterial populations. Also, using oligotyping we avoided the commonly-used 97% similarity cut-off and partitioned our dataset into homogeneous genotypic units that entail minimal phylogenetic mixture. The single-nucleotide resolution oligotyping achieves allowed us to determine various geographic patterns of bacterial and algal community structure within the confines of this isolated ecosystem. These patterns suggest different niche adaptations. As commonly observed along vertical profiles, depth played a major role in the partitioning of microbial taxa. E.g., diversity of *Nitrospina* genotypes was distinctly different in the deep samples of the shelf break area than elsewhere in the ASP. Oligotyping was also instrumental in identifying partitioning genotype diversity along horizontal gradients, e.g., in determining the diversity of diatom populations (Figure 4). These patterns suggest an abundance of niche adaptations for which selective forces and ecological implications are yet to be determined. The main observations from this study are: (1) *P. antarctica* blooms are accompanied by a unique and stable community of free-living bacteria over extended time scales and (2) *Phaeocystis* cells and colonies associate with selected bacterial taxa. We have identified taxa (e.g., SAR92) that accompany productive *Phaeocystis* populations in surface water and different taxa (e.g., *Colwellia*) that are associated with—supposedly decaying—populations of *Phaeocystis* cells at depth, below the illuminated, surface mixed layer.

Antarctic phytoplankton populations under non-bloom conditions typically include *P. antarctica* as one of the dominant species (Yager et al., 2012). Such populations in the ACC, or in coastal waters near the Antarctic Peninsula are accompanied by bacterial populations that are dominated by Proteobacteria, mostly *Pelagibacter* and SAR11-like genotypes (West et al., 2008; Brown et al., 2012; Wilkins et al., 2013a, this study). During bloom events *P. antarctica* often becomes the dominant phytoplankter, most notably in the ASP where such blooms recur annually (Arrigo et al., 1999; Smith et al., 2000; Arrigo and Van Dijken, 2003; Alderkamp et al., 2012; Yager et al., 2012; Kim et al., 2013; Dinasquet et al., submitted). Based on different proxies it has been estimated that the *P. antarctica* blooms contribute >99% of chlorophyll *a*, biomass or cell count. We found that >78% of the V6 reads for chloroplasts (a proxy for relative abundance of *Phaeocystis* cells) were contributed by *P. antarctica* in the <20-μm fraction of polynya samples. Based on chloroplast 16S-V6 we found that the bloom was dominated by a single oligotype. In adjacent waters we discovered a shift in oligotype abundances suggesting that several *Phaeocystis* genotypes did not contribute to the bloom formation in the ASP but had a distinct presence in the waters abutting the polynya.

Since the bulk of the *P. antarctica* bloom was contained in colonies >20-μm this percentage is expected to exceed 78% of the whole phytoplankton community. Whether it is by food web interactions, decay of dead *Phaeocystis* cells, viral lysis, or simple secretion of dissolved organic compounds, *Phaeocystis* blooms can have a large impact on heterotrophic activities and hence shape bacterial communities. We found that members of the Bacteroidetes were most abundant in *Phaeocystis* dominated samples. These observations are in agreement with investigations performed in other locations of the Southern Ocean (Wilkins et al., 2013a; Williams et al., 2013) and support the general standing of this phylum in the specialization of high molecular weight organic matter degradation (Thomas et al., 2011). Members of the Bacteroidetes and Proteobacteria made up 95–97% of the microbial community in the ASP bloom samples. SAR92, *Oceanospirillum-like*, and *Pelagibacter* (Proteobateria), along with *Polaribacter* *sensu lato* (Bacteroidetes) combined were 73.1% (±5.7) of the bacterial community during the 3 weeks covered by the 2010–2011 cruise. The relative abundances of these taxa are similar to those reported by Kim et al. (2013) for the later stages of the ASP *Phaeocystis* bloom (January–February 2010). In addition, these same taxa dominated the *Phaeocystis* bloom at our sampling site during the summer of 2007–2008. Based on the findings above we suggest that *P. antarctica* blooms are accompanied by stable and distinct microbial communities. Within this community we detected a single, dominant oligotype (>80% of the V6 reads) for each of the dominant taxa (e.g., SAR92, *Oceanospirillum-like*), in contrast with the multiplicity of oligotypes for taxa known from other niches (e.g., SAR86, *Nitrospina*). This observation suggests that specialized ecotypes with conserved genotype signatures co-exist (and possibly interact) with *P. antarctica*. Different phytoplankton species produce different DOM compounds, but closely related species have very similar DOM spectra (Becker et al., 2014). Consistent with this result, blooms of different* Phaeocystis* species have very similar bacterial communities associated with them (Alderkamp et al., 2007) and these bacteria readily degrade labile, presumably low molecular weight carbohydrates produced by these algae (Osinga et al., 1997; Smith et al., 1998; Janse et al., 1999). In addition, polymers excreted by *Phaeocystis* blooms provide a nitrogen rich substrate for heterotrophic bacteria (Solomon et al., 2003) and are expected to induce shifts in microbial community structure. In our study we observed that SAR86 oligotypes (Figure 5) associated with *Phaeocystis* blooms were distinct from other SAR86 in adjacent waters with diverse diatom populations as well as in the underlying deep waters. Controlled experimental manipulations and genomic analyses of bacterial metabolisms are needed to better understand the interactions between alga and bacteria and their effects on bacterial community structure.

Biomass produced by *Phaeocystis* blooms is rapidly exported to deeper waters, where cells and colonies become senescent (Ditullio et al., 2000). During the bloom in the ASP in 2010 we detected *Phaeocystis* biomass trapped beneath the surface mixed layer that provides a substrate for microbial degradation. This senescent part of the population was accompanied by a very different microbial community. Contributions by SAR92, Flavobacteriaceae and *Oceanospirillum*-like genotypes were diminished whereas the Gammaproteobacterium SAR86, *Nitrospina* and diverse members of the Verrucomicrobia had become the dominant taxa. The shift in microbial community composition toward Verrucomicrobia and Gammaproteobacteria has been reported for senescent *Phaeocystis* populations (Alderkamp et al., 2007). The increased contribution of *Nitrospina* at depth is likely a result of its role in nitrate formation from ammonium (Luecker et al., 2013) released during the decomposition of senescent *Phaeocystis* populations.

During bloom situations *Phaeocystis* is mostly found as large colonies protected by a semi-permeable membrane (see Schoemann et al., 2005, for a review). In early studies these colonies were thought of as cells within a mucopolysaccharide matrix, but this has been revised to a model where an outer membrane encloses *Phaeocystis* cells within a liquid matrix (Hamm et al., 1999). Indeed, microscopic inspection of *P. antarctica* colonies showed free-moving and rapidly swimming ciliates within the colony matrix (Delmont, unpublished data). Because of its virtually monotypic blooms and the large cells/colony size *P. antarctica* can be readily enriched by size fractionation. We showed that >3-μm fractions are significantly enriched with the Gammaproteobacteria SAR92. A preliminary estimate indicates that SAR92 and *P. antarctica* cells in surface bloom samples occur in approximately a 2:1 ratio. SAR92 is typically limited by carbon availability and despite carrying proteorhodopsin it does have a photoheterotrophic lifestyle (Stingl et al., 2007). A close association of SAR92 and *P. antarctica* (with SAR92 possibly contained within the colony liquid matrix) could thus be of mutual benefit. Preliminary findings from a metagenome analysis of the 2010-2011 ASP bloom event indicates that SAR92 and other *Phaeocystis* associated bacterial taxa may play a role in sulfur metabolism and iron acquisition via ferrochelatase and siderophore production (Delmont et al., in prep.). SAR92 was abundantly present in phytoplankton bloom samples following a natural occurrence of iron-enrichment in the Southern Ocean (West et al., 2008). This would be especially beneficial if *P. antarctica* would harbor SAR92 within its colony matrix. The fact that this matrix resembles an enclosed aqueous environment allows for rapid diffusion of these secreted compounds and hence efficient usage.

We propose that a mutualistic relationship between *Phaeocystis* and associated bacteria underpins the intensity and longevity of its blooms and thereby sequester substantial amounts of atmospheric carbon dioxide in high-latitude oceans (Smith et al., 1991). Determining the genomic content and activity of associated bacteria will help understanding these mechanisms. Conversely, we determined that a bacterial community dominated by members of the genus *Colwellia* was associated with supposedly senescent *Phaeocystis* cells at depth. Such *Colwellia* are known for the production of extracellular polysaccharides/enzyme complexes involved in the breakdown of high-molecular-weight organic compounds (Methé et al., 2005). Therefore, *Colwellia* and related taxa may play an important role in the recycling of carbon, sulfur and nitrogen by rapidly degrading bloom biomass before their complete sedimentation, a process that can last for more than 8 months (Kirchman et al., 2001b).

### Conflict of interest statement

The authors declare that the research was conducted in the absence of any commercial or financial relationships that could be construed as a potential conflict of interest.

## Supplementary material

Table S1 **Sample designations, metadata and parameters relating to the 16S-V6 sequence datasets used in the analysis underlying Figure 2**. Sequence data originated from three different projects and they are publically available on the VAMPS website (http://vamps.mbl.edu/).

Table S2 **Sample designations and environmental metadata relating to the ASPIRE cruise (2010-2011 bloom event)**.

Figure S1 **Heat map (Bray-Curtis distance) based on the relative distribution of bacterial taxa (based on GAST classification at the genus level) in 3 × 4 samples collected from the Antarctic Circumpolar Current, off the Antarctica peninsula and inside the Amundsen Sea polynya**. Dissimilarity between samples is reflected by a color gradient ranging from red (high dissimilarity) to blue (low dissimilarity).

Figure S2 **Correspondence analysis (COA, euclidean distance) of the relative distribution of major bacterial taxa across high throughput sequencing datasets from the Antarctic Circumpolar Current, off the Antarctic Peninsula and the Amundsen Sea polynya**. The latter includes surface and deep bacterial communities sampled at the outer rim of the polynya, near the Dotson Glacier and in the open waters of the polynya. Note that the “polynya deep” label is partly covering the “Dotson Glacier deep” label. Pyrosequencing datasets of the Amundsen Sea polynya representing the 0.2–3 μm size fraction sampled during the 2007–2008 bloom event (4 surface and 3 deep samples; see Table S1) were included in the analysis.

Figure S3 **(A)** represents the number of bacterial species identified in each V6 data from the 2010–2011 bloom event using the Global Assignment of Sequence Taxonomy (GAST) pipeline (Huse et al., 2008) and the SILVA 111 database for reference (Quast et al., 2013). **(B)** represents the number of reads generated for each data set. Datasets represent surface (*n* = 18) and deep samples (*n* = 5).

Figure S4 **Principal component analysis (PCA) of the relative abundance of major bacterial taxa (based on GAST classification) in the <3 μm (A) and >3 μm (B) size fractions of surface and deep samples of the Amundsen polynya during a *Phaeocystis* bloom in 2007–2008 by comparing free living bacteria (0.2–3-μm size fraction, ***n*** = **4**) and alga/particulate associated bacteria (>3-μm size fraction, ***n*** = **4**)**.
