# Supplementary material for: Phaeocystis antarctica blooms strongly influence bacterial community structures in the Amundsen Sea polynya
Source: Front Microbiol. 2014 Dec 19;5:646. doi: 10.3389/fmicb.2014.00646 (PMC4271704; doi:10.3389/fmicb.2014.00646)
Supplement: Table S1 — Sample designations, metadata and parameters relating to the 16S-V6 sequence datasets used in the analysis underlying Figure 2. Sequence data originated from three different projects and they are publically available on the VAMPS website (http://vamps.mbl.edu/). [file Table1.PDF]

	Sample name	Location	Sample depth	Sampling date	Filtration strategy	GPS Location		Number of reads	Sea floor depth
Cruise 2010	AFP_Ant5	Polynya open water	Surface	21_12_2010	0.2 - 20 microns	072 59.917S	113 18.556W	1.095.500	439
	AFP_Ant8	Polynya open water	Surface	02_01_2011	0.2 - 20 microns	073 38.9172S	113 13.3290W	397.682	653
	AFP_Ant9	Polynya open water	Surface	03_01_2011	0.2 - 20 microns	073 36.13S	113 08.91W	409.697	640
	AFP_Ant11	Polynya open water	Surface	24_12_2010	0.2 - 20 microns	072 57.7798S	115 45.6638W	374.789	680
	AFP_Ant12	Polynya open water	Surface	28_12_2010	0.2 - 20 microns	073 42.0776S	115 26.9948W	618.393	940
	AFP_Ant13	Polynya open water	Surface	29_12_2010	0.2 - 20 microns	073 24.9694S	115 15.0156W	361.133	1055
	AFP_Ant14	Polynya open water	Surface	01_01_2011	0.2 - 20 microns	073 42.437S	113 15.919W	376.002	803
	AFP_Ant15	Polynya open water	Surface	19_12_2010	0.2 - 20 microns	073 34.243S	112 40.080W	2.166.926	551
	AFP_Ant16	Polynya open water	394 meters	22_12_2010	0.2 - 20 microns	073 07.249S	111 59.936W	253.031	406
	AFP_Ant17	Polynya open water	890 meters	24_12_2010	0.2 - 20 microns	073 23.8683S	114 50.50517W	474.761	892
	AFP_Ant18	Polynya open water	Surface	31_12_2010	0.2 - 20 microns	073 48.0794S	113 09.9911W	598.915	746
	AFP_Ant19	Polynya open water	Surface	05_01_2011	0.2 - 20 microns	072 44.450S	116 01.183W	507.085	660
	AFP_Ant20	Polynya open water	Surface	22_12_2010	0.2 - 20 microns	74 07.249S	112 59.936W	576.916	406
	AFP_Ant21	Polynya open water	Surface	26_12_2010	0.2 - 20 microns	073 16.7729S	112 06.2453W	500.601	431
	AFP_Ant22	Polynya open water	Surface	23_12_2010	0.2 - 20 microns	073 21.0260S	114 07.6117W	597.005	737
	AFP_Ant1	Shelf Break	Surface	08_01_2011	0.2 - 20 microns	071 34.139S	113 00.574W	260.558	597
	AFP_Ant2	Shelf Break	820 meters	08_01_2011	0.2 - 20 microns	071 51.4137S	118 16.7921W	379.674	830
	AFP_Ant3	Shelf Break	591 meters	08_01_2011	0.2 - 20 microns	071 34.139S	113 00.574W	302.983	597
	AFP_Ant10	Shelf Break	Surface	08_01_2011	0.2 - 20 microns	071 51.4137S	118 16.7921W	288.636	830
	AFP_Ant4	Dotson ice shelf	910 meters	18_12_2010	0.2 - 20 microns	074 13.895S	112 21.498W	114.996	915
	AFP_Ant6	Dotson ice shelf	Surface	18_12_2010	0.2 - 20 microns	074 13.895S	112 21.498W	253.731	915
	AFP_Ant7	Dotson ice shelf	Surface	17_12_2010	0.2 - 20 microns	074 13.183S	112 0.110W	400.578	870
	AFP_Ant23	Dotson ice shelf	Surface	18_12_2010	0.2 - 20 microns	074 12.544S	112 30.426W	384.641	1063
Cruise 2007	ASA_001	Polynya open water	Surface	18_12_2007	0.2 - 3 microns	073 94 S	115 68 W	14.635	-
	ASA_003	Polynya open water	20 meters	18_12_2007	0.2 - 3 microns	073 94 S	115 68 W	22.562	-
	ASA_005	Polynya open water	35 meters	18_12_2007	0.2 - 3 microns	073 94 S	115 68 W	12.987	-
	ASA_008	Polynya open water	100 meters	18_12_2007	0.2 - 3 microns	073 94 S	115 68 W	12 560	-
	ASA_010	Polynya open water	250 meters	18_12_2007	0.2 - 3 microns	073 94 S	115 68 W	17.244	-
	ASA_014	Polynya open water	500 meters	18_12_2007	0.2 - 3 microns	073 94 S	115 68 W	30.614	-
	ASA_013	Polynya open water	780 meters	18_12_2007	0.2 - 3 microns	073 94 S	115 68 W	24.905	-
	ASA_002	Polynya open water	Surface	18_12_2007	> 3 microns	073 94 S	115 68 W	37.776	-
	ASA_004	Polynya open water	20 meters	18_12_2007	> 3 microns	073 94 S	115 68 W	31.928	-
	ASA_006	Polynya open water	35 meters	18_12_2007	> 3 microns	073 94 S	115 68 W	33.224	-
	ASA_009	Polynya open water	100 meters	18_12_2007	> 3 microns	073 94 S	115 68 W	21.103	-
	ASA_011	Polynya open water	250 meters	18_12_2007	> 3 microns	073 94 S	115 68 W	13.751	-
Southern Ocean	PAL-3-1B	Antarctic circumpolar current	Surface	05_01_2008	> 0.2 microns	066 86 S	063 97 W	30.684	-
	PAL-3-2B	Antarctic circumpolar current	Surface	12_01_2008	> 0.2 microns	066 86 S	063 97 W	2.351	-
	PAL-5-1B	Antarctic circumpolar current	Surface	12_01_2008	> 0.2 microns	073 03 S	066 45 W	3.331	-
	PAL-5-2B	Antarctic circumpolar current	Surface	12_01_2008	> 0.2 microns	073 03 S	066 45 W	14.322	-
	PAL-4-1B	Antarctic circumpolar current	100 meters	12_01_2008	> 0.2 microns	066 86 S	063 97 W	20.158	-
	PAL-4-2B	Antarctic circumpolar current	100 meters	12_01_2008	> 0.2 microns	066 86 S	063 97 W	21.51	-
	PAL-6-1B	Antarctic circumpolar current	120 meters	23_01_2008	> 0.2 microns	073 03 S	066 45 W	9.88	-
	PAL-6-2B	Antarctic circumpolar current	120 meters	23_01_2008	> 0.2 microns	073 03 S	066 45 W	14.972	-
	PAL_1_1B	Antarctic Peninsula	Surface	05_01_2008	> 0.2 microns	064 41 S	064 94 W	6.287	-
	PAL_1_2B	Antarctic Peninsula	Surface	05_01_2008	> 0.2 microns	064 41 S	065 94 W	5.033	-
	PAL_7_1B	Antarctic Peninsula	Surface	27_01_2008	> 0.2 microns	069 62 S	067 90 W	9000	-
	PAL_7_2B	Antarctic Peninsula	Surface	27_01_2008	> 0.2 microns	069 62 S	067 90 W	4070	-

Table S1
