# Supplementary figures and images for: Phaeocystis antarctica blooms strongly influence bacterial community structures in the Amundsen Sea polynya
Source: Front Microbiol. 2014 Dec 19;5:646. doi: 10.3389/fmicb.2014.00646 (PMC4271704; doi:10.3389/fmicb.2014.00646)

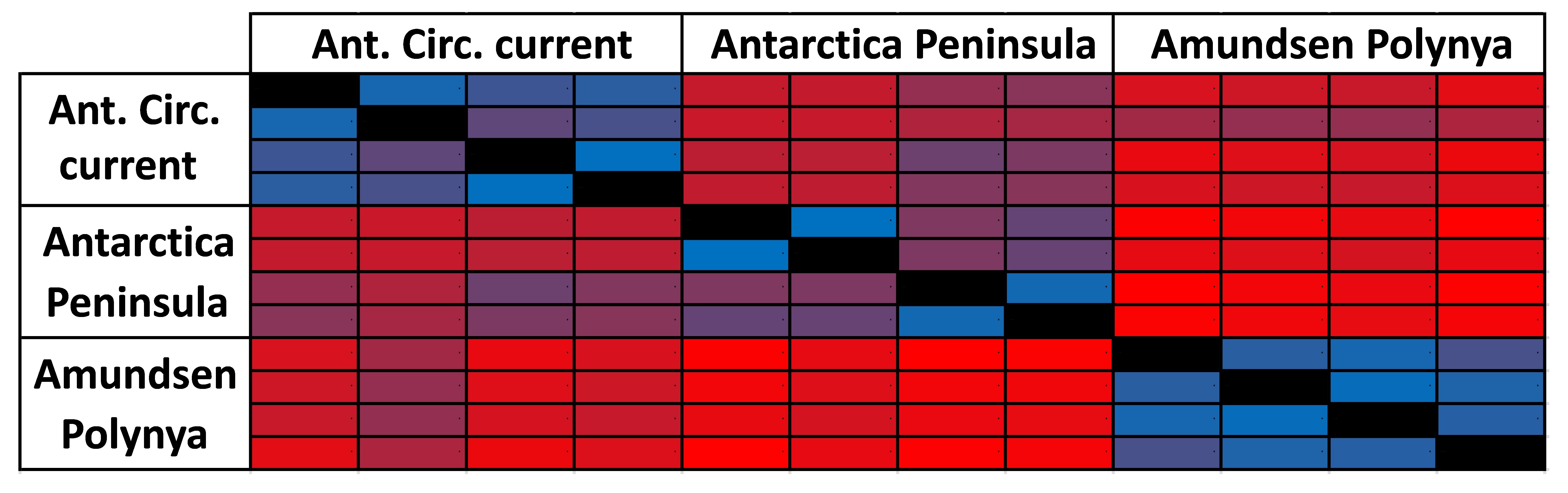

Supplement: Figure S1 — Heat map (Bray-Curtis distance) based on the relative distribution of bacterial taxa (based on GAST classification at the genus level) in 3 × 4 samples collected from the Antarctic Circumpolar Current, off the Antarctica peninsula and inside the Amundsen Sea polynya. Dissimilarity between samples is reflected by a color gradient ranging from red (high dissimilarity) to blue (low dissimilarity). [file Image1.JPEG]

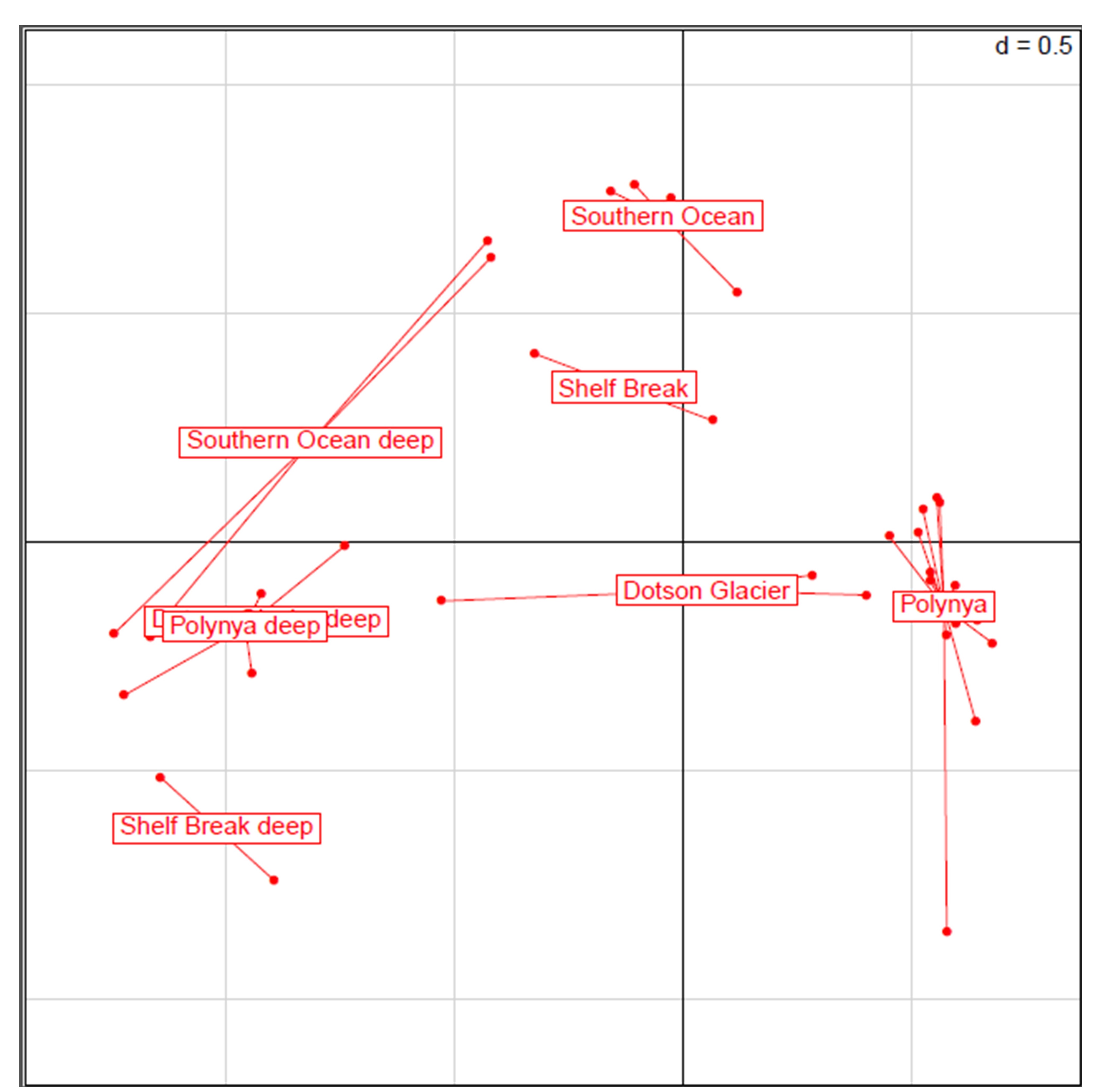

Supplement: Figure S2 — Correspondence analysis (COA, euclidean distance) of the relative distribution of major bacterial taxa across high throughput sequencing datasets from the Antarctic Circumpolar Current, off the Antarctic Peninsula and the Amundsen Sea polynya. The latter includes surface and deep bacterial communities sampled at the outer rim of the polynya, near the Dotson Glacier and in the open waters of the polynya. Note that the “polynya deep” label is partly covering the “Dotson Glacier deep” label. Pyrosequencing datasets of the Amundsen Sea polynya representing the 0.2–3 μm size fraction sampled during the 2007–2008 bloom event (4 surface and 3 deep samples; see Table S1) were included in the analysis. [file Image2.JPEG]

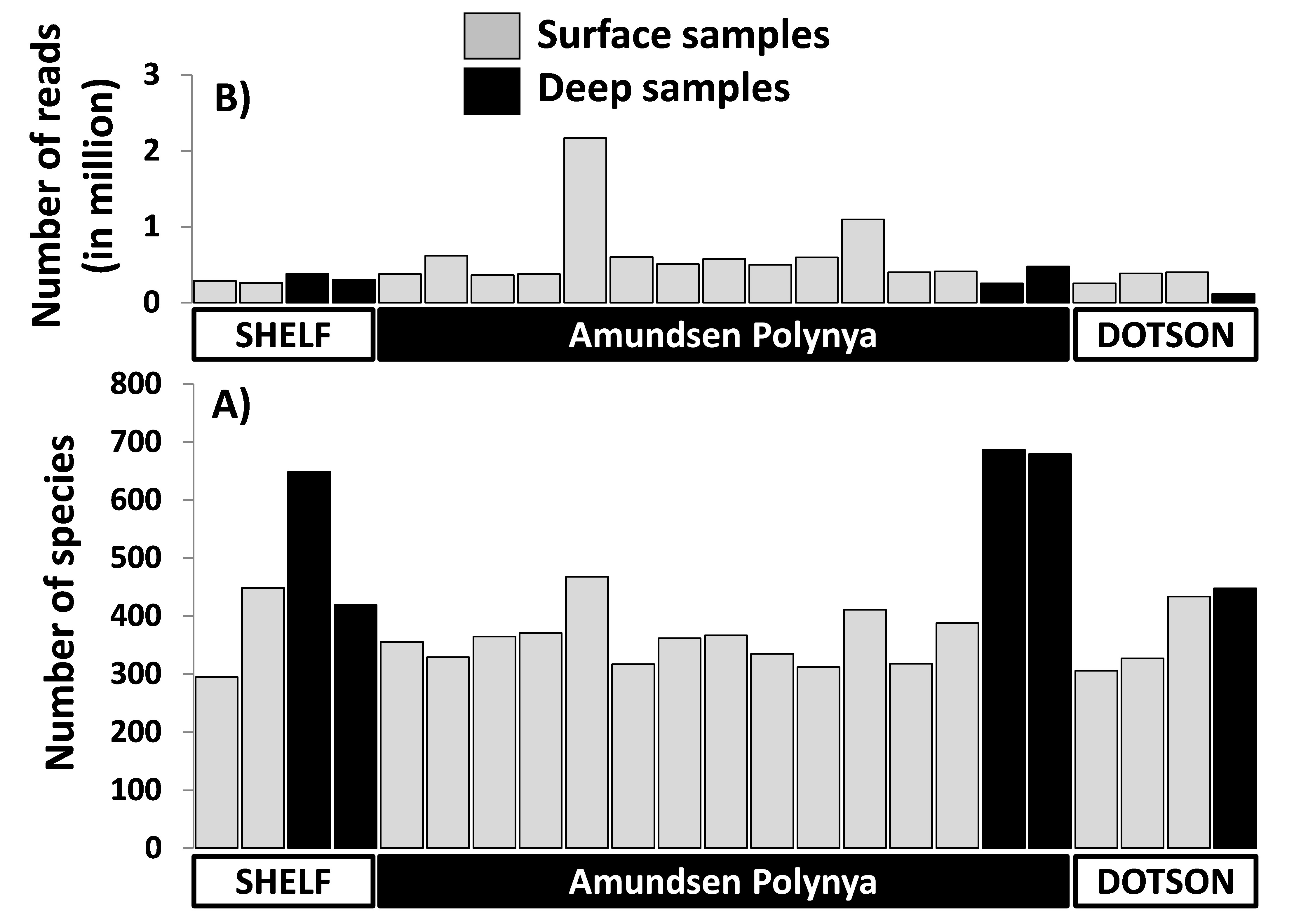

Supplement: Figure S3 — (A) represents the number of bacterial species identified in each V6 data from the 2010–2011 bloom event using the Global Assignment of Sequence Taxonomy (GAST) pipeline (Huse et al., 2008) and the SILVA 111 database for reference (Quast et al., 2013). (B) represents the number of reads generated for each data set. Datasets represent surface (n = 18) and deep samples (n = 5). [file Image3.JPEG]

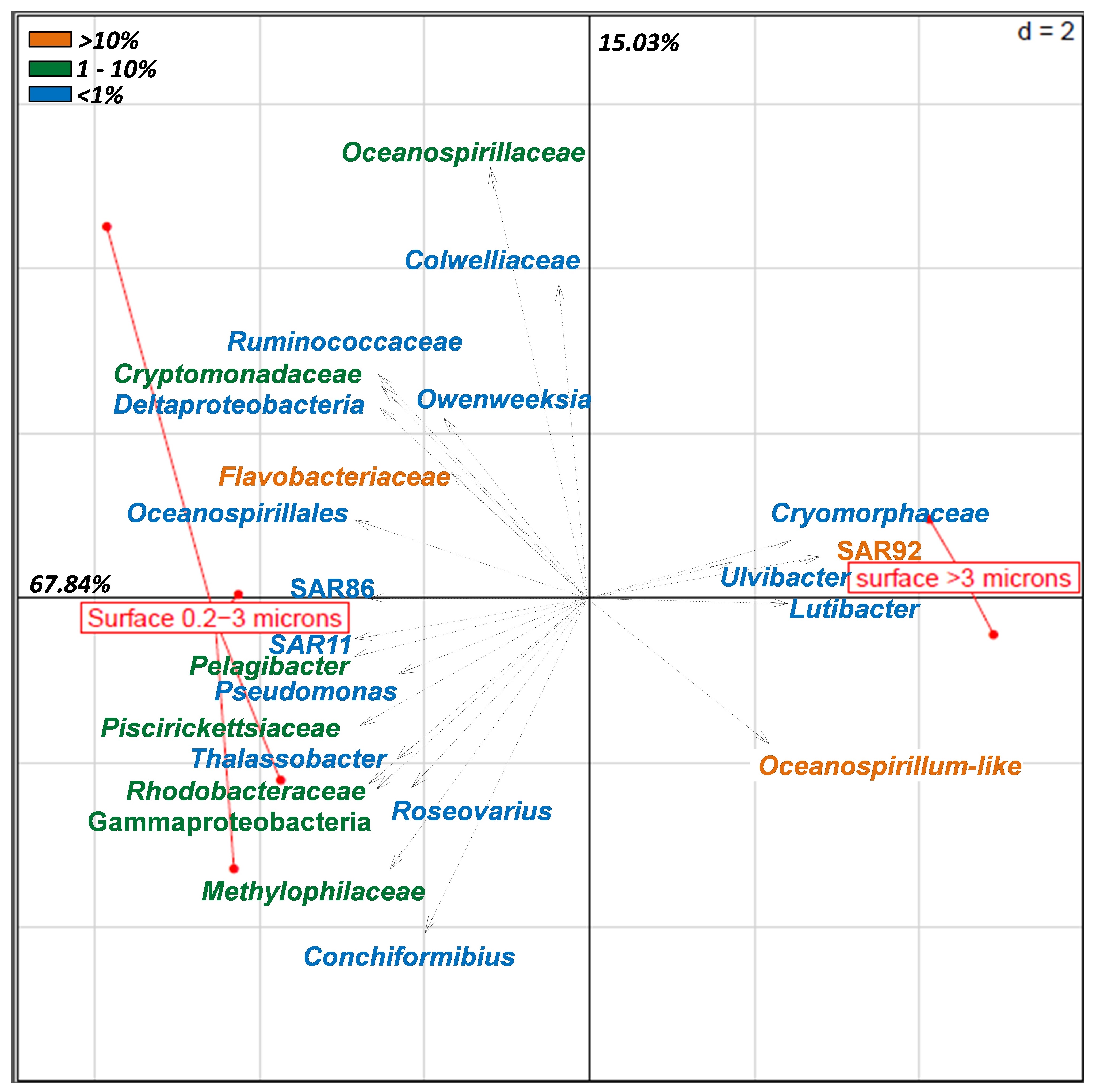

Supplement: Figure S4 — Principal component analysis (PCA) of the relative abundance of major bacterial taxa (based on GAST classification) in the <3 μm (A) and >3 μm (B) size fractions of surface and deep samples of the Amundsen polynya during a Phaeocystis bloom in 2007–2008 by comparing free living bacteria (0.2–3-μm size fraction, n = 4) and alga/particulate associated bacteria (>3-μm size fraction, n = 4). [file Image4.JPEG]
